# Supplementary material for: A viral protease relocalizes in the presence of the vector to promote vector performance
Source: Nat Commun. 2017 Feb 16;8:14493. doi: 10.1038/ncomms14493 (PMC5316897; doi:10.1038/ncomms14493)
Supplement: Supplementary Information — Supplementary Figures and Supplementary Tables [file ncomms14493-s1.pdf]

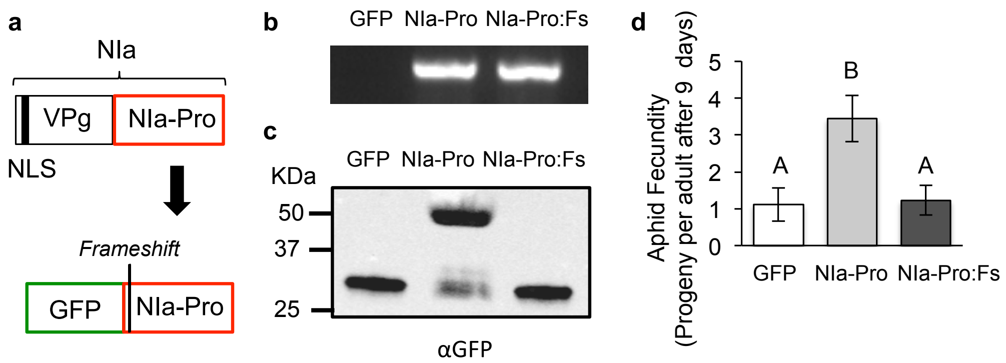

Supplementary Figure 1. Production of the TuMV protein NIa-Pro is required to increase aphid fecundity. (a) The figure shows the frameshift mutant of GFP:NIa-Pro used in the study. (b) A PCR was performed to measure NIa-Pro transcript abundance in plants agroinfiltrated with free GFP, GFP:NIa-Pro and GFP:NIa-Pro with a frameshift (NIa-Pro:Fs) which inhibits the expression of the protein. (c) The image represents a western blot from leaves expressing free GFP, GFP:NIa-Pro and GFP:NIa-Pro:Fs incubated with a GFP antibody. The NIa-Pro protein is not produced with the frameshift mutant. (d) Number of progeny produced by aphids on *N. benthamiana* agroinfiltrated with free GFP, GFP:NIa-Pro, or with GFP:NIa-Pro:Fs. Mean +/- standard error (SE); N = 12, letters represent significant differences, ANOVA and Tukey HSD post hoc, $P < 0.05$. Results from one out of three independent experiments are displayed.

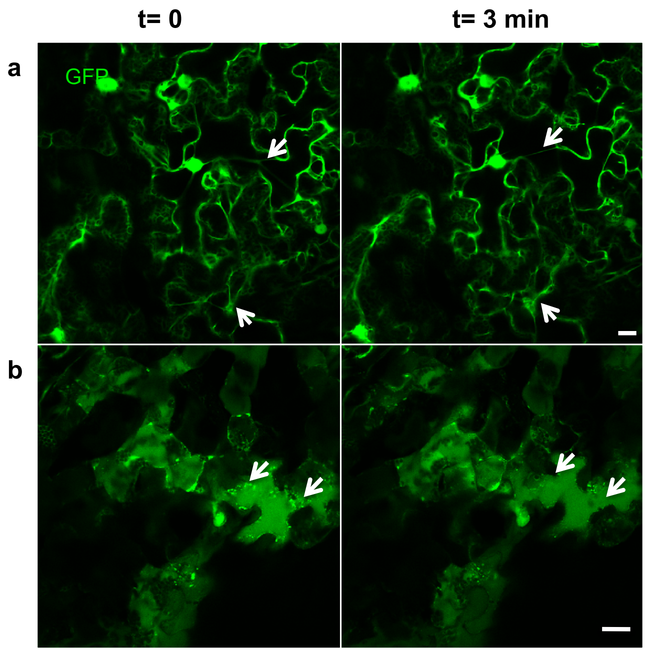

Supplementary Figure 2. Time lapse of the cytoplasmic streaming and vacuole trafficking. Images show confocal time series of a single z plane of *N. benthamiana* leaves agroinfiltrated with (a) free GFP and (b) GFP:NIa-Pro. Images show the cytoplasmic streaming (a) and the intra-vacuole trafficking (b) with dots, aggregates or filaments appearing or disappearing (white arrows). Scale bars = 20 μm.

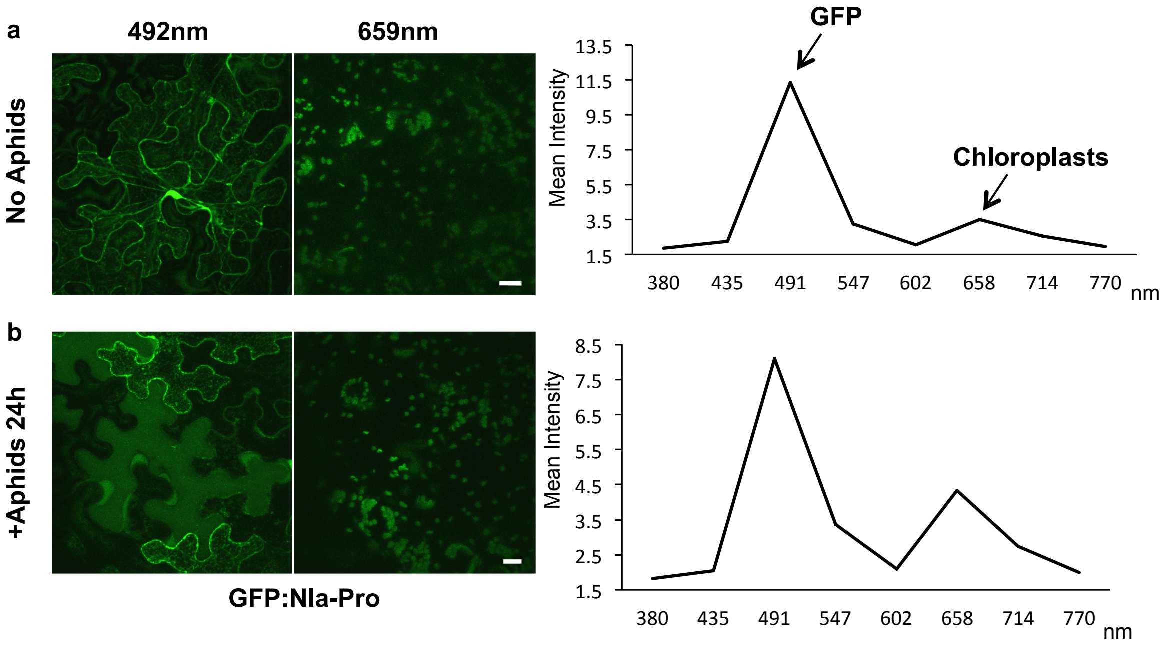

Supplementary Figure 3. Emission spectrum of GFP:NIa-Pro. Images show confocal projections of *N. benthamiana* leaves agroinfiltrated with GFP:NIa-Pro (a) without aphids or (b) with aphids present. The graphs on the left show a scan across 380-800nm. The biggest peak corresponding to the GFP emission is characteristic of the phenotypes described in the paper (images on left, emission at 492nm) and the small peak corresponds to the chloroplast's autofluorescence (images on right, emission at 659nm). Scale bars = 20 µm.

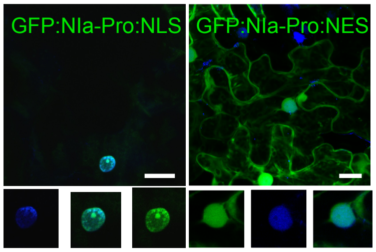

Supplementary Figure 4. NIa-Pro localization mutants. Images show confocal projection of *N. benthamiana* leaves agroinfiltrated with GFP:NIa-Pro:NLS (left panel) and GFP:NIa-Pro:NES (right panel) and stained with DAPI in blue. Single sections are shown in the small panels. Scale bars = 20 µm. NLS = Nuclear localization signal. NES = Nuclear export signal.

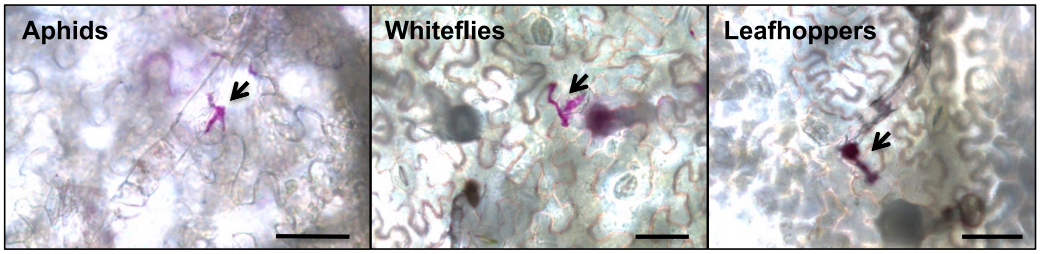

Supplementary Figure 5. Acid Fuchsin stain of *Nicotiana benthamiana* leaves infested with different insects. Images show the saliva sheaths of the different insects (black arrows) demonstrating that the insects probe on the leaves . Scale bars = 50 µm.

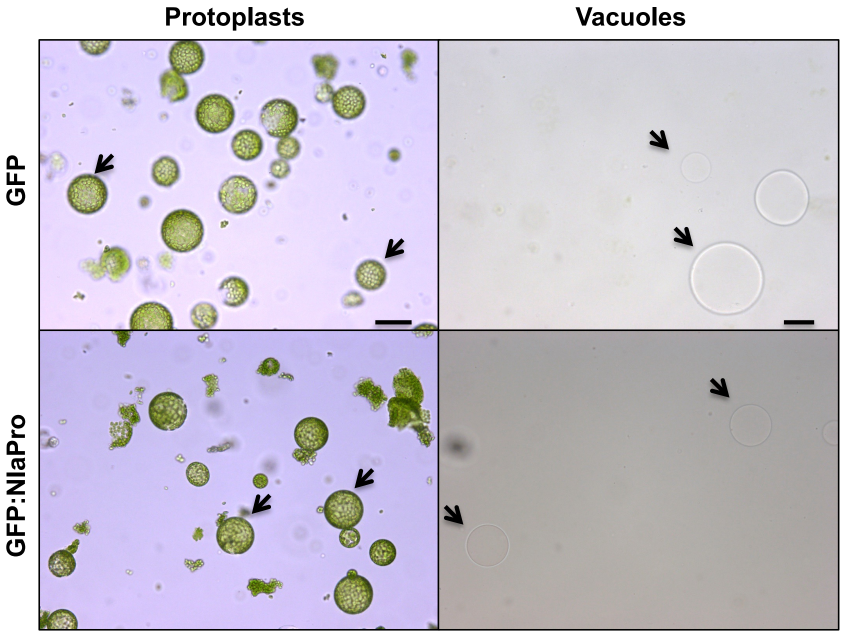

Supplementary Figure 6. Vacuole purification of *N. benthamiana* observed under the microscope. Images show the protoplast preparation on the left and the vacuole purification from the protoplasts on the right. Scale bars = 50 µm.

Fig.3c

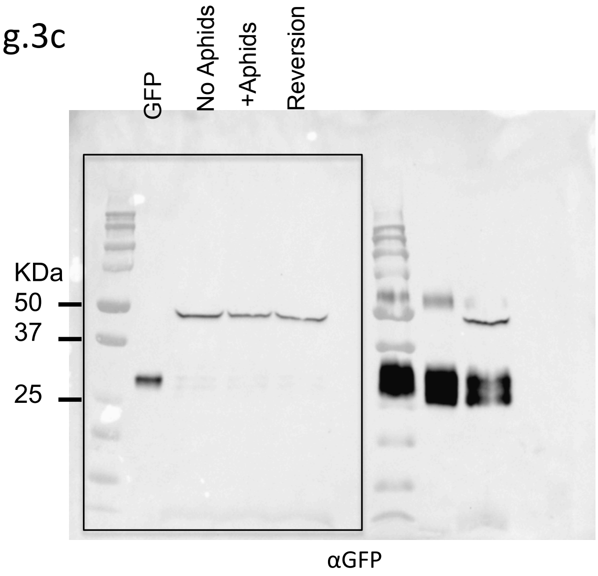

Fig.5c

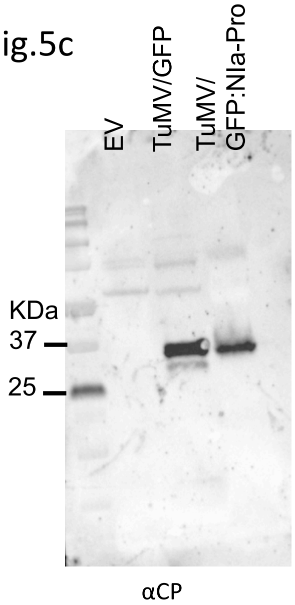

Fig.5f

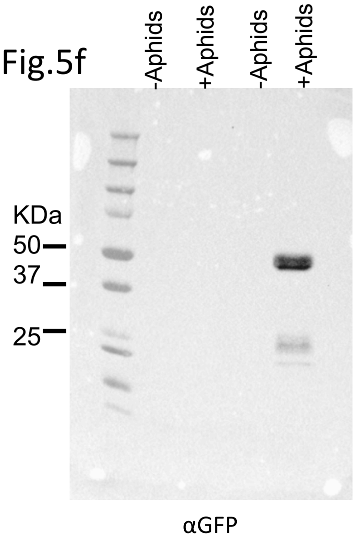

Fig.5f

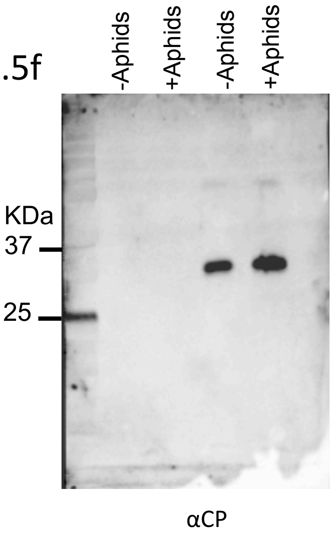

Supplementary Figure 7. Uncropped scans of the Western blots shown in the indicated figures. The cropped region for the Figure 3c is indicated by a black rectangle. The antibodies used are indicated.

Fig.7e

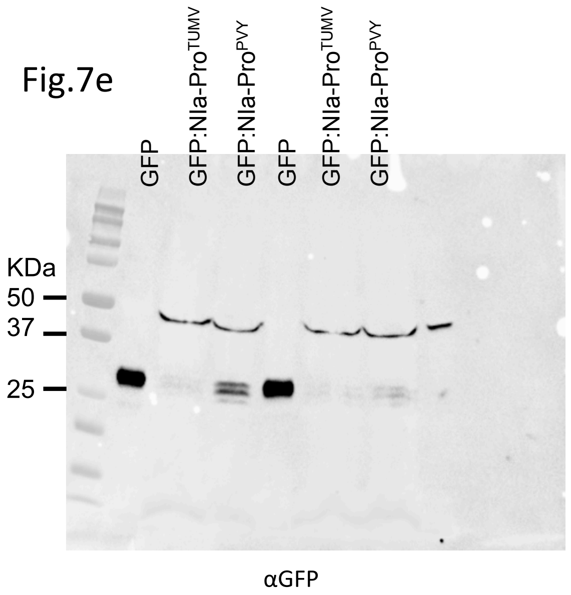

Supplementary.1b

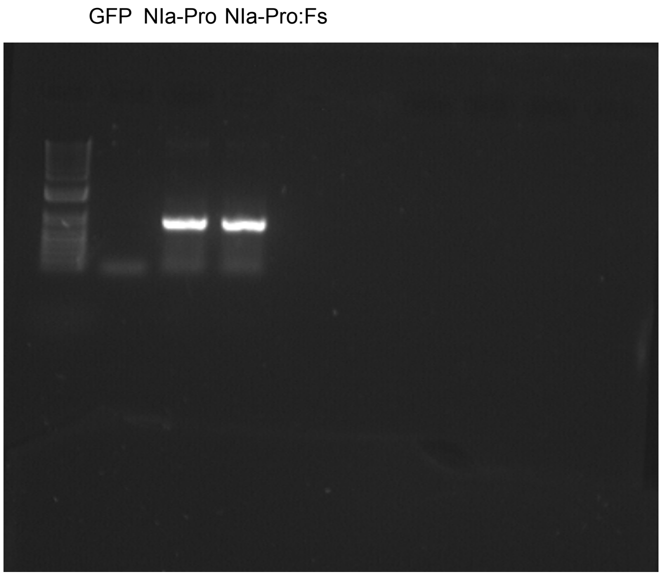

Supplementary.1c

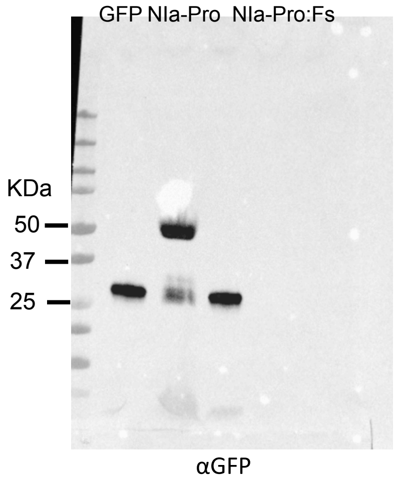

Supplementary Figure 8. Uncropped scans of the Western blots shown in the indicated figures. The antibodies used are indicated.

Primers names	Primers sequences
GtwNIaF	5'-GGGGACAAGTTTGTACAAAAAAGCAGGCTTCATGAGTAACTCCATGTTCAGAGGGTTG-3'
GtwNIaR	5'-GGGGACCACTTTGTACAAGAAAGCTGGGTCCTATTGTGCGTAGACTGCCGT-3'
GtwNIaNLS-R	5'-GGGGACCACTTTGTACAAGAAAGCTGGGTCCTATACCTTTCTCTTCTTTTTTGGTTGTGCGTAGACTGCCGTGC-3'
GtwNIa:Fs-F	5'-GGGGACAAGTTTGTACAAAAAAGCAGGCTTCATGTAGTAACTCCATGTTCAGAGGGTTG-3'
GtwNIaNES-R	5'-CTATTTGTTAATATCTAGACCAGCCAGCTTCAGGGCCAGTTCGTTTTGTGCGTAGACTGCCGT-3'
SacII-eGFP-F	5’-AATGCCGCGGGAATGGTGAGCAAGGGCGAGG-3’
NIaPro-SacII-R	5’-ATTTCCCGCGGCTTGTGCGTAGACTGCCGTGCCTAAAGTAAGCTCGCATCTTGTACAGCTCGTC-3’
GtwNIa(PVY)F	5’-GGGGACAAGTTTGTACAAAAAAGCAGGCTTCATGGCTAAATCGCTCATGAGAGGC-3’
GtwNIa(PVY)R	5’-GGGGACCACTTTGTACAAGAAAGCTGGGTCCTATTGCTCCACCACTACATCATG-3’

TABLE 1. Primers sequences used for the cloning.
